# MRSA Nasal Carriage Patterns and the Subsequent Risk of Conversion between Patterns, Infection, and Death

**DOI:** 10.1371/journal.pone.0053674

**Published:** 2013-01-10

**Authors:** Kalpana Gupta, Richard A. Martinello, Melissa Young, Judith Strymish, Kelly Cho, Elizabeth Lawler

**Affiliations:** 1 The National Center for Occupational Health and Infection Control (COHIC), Office of Public Health, Department of Veterans Affairs, Gainesville, Florida, United States of America; 2 Department of Medicine, Veterans Affairs Boston Health Care System, West Roxbury, Massachusetts, United States of America; 3 Massachusetts Veterans Epidemiology Research and Information Center, Jamaica Plain, Massachusetts, United States of America; 4 Department of Medicine, Boston University School of Medicine, Boston, Massachusetts, United States of America; 5 Harvard Medical School, Boston, Massachusetts, United States of America; 6 Veterans Health Administration, Office of Public Health, Washington, D. C., United States of America; 7 Departments of Medicine and Pediatrics, Yale School of Medicine, New Haven, Connecticut, United States of America; Vanderbilt University, United States of America

## Abstract

**Background:**

Patterns of methicillin-resistant *S. aureus* (MRSA) nasal carriage over time and across the continuum of care settings are poorly characterized. Knowledge of prevalence rates and outcomes associated with MRSA nasal carriage patterns could help direct infection prevention strategies. The VA integrated health-care system and active surveillance program provides an opportunity to delineate nasal carriage patterns and associated outcomes of death, infection, and conversion in carriage.

**Methods/Findings:**

We conducted a retrospective cohort study including all patients admitted to 5 acute care VA hospitals between 2008–2010 who had nasal MRSA PCR testing within 48 hours of admission and repeat testing within 30 days. The PCR results were used to define a baseline nasal carriage pattern of never, intermittently, or always colonized at 30 days from admission. Follow-up was up to two years and included acute, long-term, and outpatient care visits. Among 18,038 patients, 91.1%, 4.4%, and 4.6% were never, intermittently, or always colonized at the 30-day baseline. Compared to non-colonized patients, those who were persistently colonized had an increased risk of death (HR 2.58; 95% CI 2.18;3.05) and MRSA infection (HR 10.89; 95% CI 8.6;13.7). Being in the non-colonized group at 30 days had a predictive value of 87% for being non-colonized at 1 year. Conversion to MRSA colonized at 6 months occurred in 11.8% of initially non-colonized patients. Age >70 years, long-term care, antibiotic exposure, and diabetes identified >95% of converters.

**Conclusions:**

The vast majority of patients are not nasally colonized with MRSA at 30 days from acute hospital admission. Conversion from non-carriage is infrequent and can be risk-stratified. A positive carriage pattern is strongly associated with infection and death. Active surveillance programs in the year following carriage pattern designation could be tailored to focus on non-colonized patients who are at high risk for conversion, reducing universal screening burden.

## Introduction

Nasal colonization with *S. aureus* is a dynamic process, with gain and loss of carriage associated with a combination of factors [1]. Once a person becomes colonized with *S. aureus*, the risk of infection increases and remains persistently elevated [2], [3], [4], [5]. Grossly, *S. aureus* carriage can be divided into patterns of never colonized, always colonized, or intermittently colonized [6]. The distribution of these patterns is relatively well established for methicillin-sensitive *S. aureus* (MSSA) but not for methicillin-resistant *S. aureus* (MRSA), particularly in the era of community MRSA. A number of hospitals and other health care facilities in the US test patients for nasal MRSA colonization, however, most only assess carriage when patients are admitted to an intensive care unit. Thus, changes in colonization during hospitalization, in the community, and subsequent hospitalization at another facility are not detected [7]. Understanding the natural history of MRSA carriage patterns over time as well as the associated risks of infection and death is important for directing infection prevention activities such as active surveillance and decolonization programs [8].

The Department of Veterans Affairs (VA) has been performing active surveillance for nasal MRSA carriage since late 2007. Unique features of this program include multiple assessments during a single hospitalization (admission, unit transfer, and discharge) and a high patient retention rate, as the majority of veterans return to the VA integrated health-care system for care. A recent report demonstrated reductions in MRSA infections on a national level in association with this program, but did not report individual risk-adjusted patient-level outcomes [9]. The current study aims to characterize patterns of nasal MRSA carriage among a large cohort of individual patients and evaluate associated risks for conversion, infection, and death. We demonstrate that the majority of hospitalized patients are non-carriers of nasal MRSA and the small proportion likely to convert to a colonized state can be identified by clinical criteria, suggesting screening programs can be reduced in scope to focus on these patients. Patients who are colonized have significantly elevated risks of MRSA infection and death compared to non-colonized patients, even after adjusting for health care exposure and co-morbidities.

## Methods

### Ethics Statement

VA Boston IRB approval was obtained prior to data extraction and analysis. As per IRB approval, written informed consent was waived as this was a retrospective database only study utilizing the existing electronic health record and the research would not be practicable without the waiver.

### Study Population

We conducted a retrospective cohort study of patients admitted to any of the 5 acute care hospitals in the New England Veterans Integrated Service Network (VISN 1) between January 2008 and December 2010. VISN 1 consists of 1,084 inpatient beds and cares for approximately 240,000 unique patients annually in the states of Maine, New Hampshire, Vermont, Rhode Island, Massachusetts, and Connecticut. Data from the acute care hospitals, 3 long term care facilities, and all associated VA outpatient community clinics in VISN 1 were captured.

All patients who had an acute care admission, a nasal PCR screening test for MRSA performed within 48 hours of the admission (index admission), and at least one more nasal screen performed within the next 30 days were eligible for study inclusion. Index admissions were censored at 6/30/2010 to allow a minimum of 6 months of follow-up time. Patients were followed for a maximum of 2 years, or until the date of death, a gap of 18 months without VA health care use, or the study end date (12/31/2010).

### Carriage Pattern Definitions

The GeneXpert MRSA PCR assay (Cepheid, Sunnyvale, CA) is used to test for nasal MRSA carriage at each hospital. Nasal carriage patterns were designated as never (no nasal swabs positive), always (>80% of nasal swabs positive), or intermittent (>1 but less than 80% of swabs positive) and were determined on the basis of all available nasal PCR test results within the first 30 day period of the index admission. The carriage patterns were determined during two different time periods. The first used all screen results from the first 30-days. In order to account for the changes in carriage pattern over the study period, a second carriage pattern designation was determined using all screen results from the entire follow-up window (dynamic carriage pattern). Patients were removed from the never colonized group and excluded from the study if they had a positive clinical culture for MRSA in the previous year. Outcomes were assessed for both the 30-day carriage pattern as well as the dynamic carriage pattern. We performed a validation of the 30-day PCR pattern rule by evaluating concordance with carriage patterns in patients who had 5 or more swabs within a 1 year period (including the swabs performed in the first 30 days) because a rule for determining *S. aureus* carriage pattern has not been validated using PCR [10].

### Data Collection

The electronic health record was used to capture antibiotic and health-care exposures, co-morbidities, and outcomes. Clinical cultures for MRSA and MSSA were extracted from the VISN 1 data warehouse. Any positive culture from blood or bone was coded as infection. Positive cultures from wounds, skin, sputum, urine or other non-sterile sites were coded as infection if there was an active antimicrobial agent administered within 5 days of the culture result. Data from all inpatient and outpatient encounters were included.

### Statistical Analysis

Baseline characteristics are described by frequencies and proportions stratified by the 30-day carriage pattern designation. Chi-squared tests were used to evaluate clinical parameters among patients in each of the three carriage pattern groups. A multi-variable logistic regression was used to assess the effects of multiple potential risk factors on the three categories of colonization status, as well as to determine what risk factors predicted conversion to MRSA colonized status during the six month period following initial screening.

Cox proportional hazards models were used to assess time to each of the three outcomes (death, first MRSA infection, and first MSSA infection) among the three carriage groups. The models were run twice for each outcome to allow a comparison of the 30-day carriage pattern and the dynamic carriage pattern over the study period. Cumulative incidence curves were generated to plot the resulting survival for each outcome. SAS statistical software was used for all programming with p-values of < 0.05 as statistically significant.

## Results

**Figure 1:**
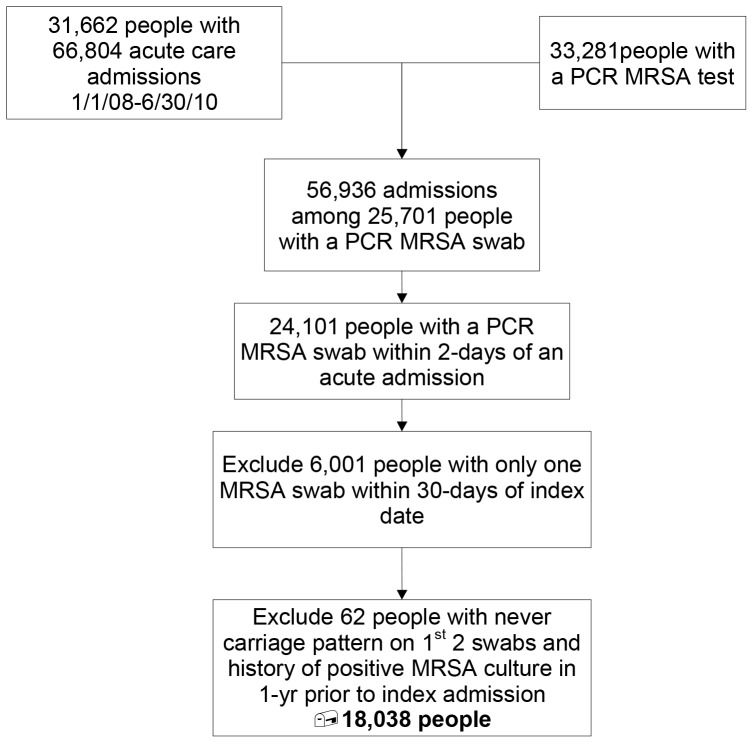
Flow Diagram. Derivation of the study cohort.

A total of 31,662 unique patients had 66,804 hospitalizations to at least one of the 5 acute care hospitals over the 3-year study period. A nasal MRSA PCR result was available within 2 days of the index admission in 24,101 (76%) patients, and two or more MRSA surveillance results were available over a 30 day period in 18,100 patients (Figure 1). Sixty-two patients were excluded because they had a non-colonized carriage pattern but a positive culture in the previous year, leaving 18,038 patients in the study cohort. Consistent with the overall VISN 1 patient population, 96% of subjects were male and the mean age was 66.7 years (Table 1). Among the 13,664 patients for whom race was recorded, 12,466 (91.2%) were white and 7.9% were black.

**Table 1:** Baseline Characteristics for Study Cohort by 30-Day Carriage Pattern.

Baseline Characteristic	All	Carriage Pattern During First 30-Days		
	N = 18,038n (%)	Nevern = 16,425 (91.1)n (%)	Intermittentn = 793 (4.4)n (%)	Alwaysn = 820 (4.6)n (%)
***Demographics***				
Male Sex	17,259 (95.7)	15,690 (95.5)	771 (97.2)	798 (97.3)
Age, Years, 71+	7,211 (40.0)	6,397 (39.0)	374 (47.2)	440 (53.7)
Race: White	12,466 (69.1)	11,273 (68.6)	561 (70.7)	632 (77.1)
Black	1,078 (6.0)	984 (6.0)	49 (6.2)	45 (5.5)
Other	120 (0.67)	108 (0.66)	<11 (0.76)	<11 (0.73)
Missing	4,374 (24.3)	4,060 (24.7)	177 (22.3)	137 (16.7)
***Health Care Exposure, Previous Year***				
Acute Care Admission	3,348 (18.6)	2,820 (17.2)	208 (26.2)	320 (39.0)
Long Term Care Admission	570 (3.2)	410 (2.5)	53 (6.7)	107 (13.1)
Outpatient Procedure	8,949 (49.6)	8,116 (49.4)	400 (50.4)	433 (52.8)
Inpatient Procedure	309 (1.7)	250 (1.5)	17 (2.1)	42 (5.1)
Dialysis	83 (0.46)	71 (0.43)	<11 (0.25)	<11 (1.2)
***S. aureus cultures, Previous Year***				
MRSA Clinical Culture	104 (0.58)	0 (0.0)	28 (3.5)	76 (9.3)
MSSA Clinical Culture	224 (1.2)	190 (1.2)	16 (2.0)	18 (2.2)
***Co-morbidities, Previous Year***				
Malignancy	3,276 (18.2)	2,940 (17.9)	170 (21.4)	166 (20.2)
Chronic lung disease	437 (2.4)	379 (2.3)	24 (3.0)	34 (4.2)
Diabetes mellitus	5,019 (27.8)	4,465 (27.2)	252 (31.8)	302 (36.8)
Renal disease	1,058 (5.9)	878 (5.4)	70 (8.8)	110 (13.4)
Liver disease	454 (2.5)	395 (2.4)	25 (3.2)	34 (4.2)
Skin disease	902 (5.0)	806 (4.9)	36 (4.5)	60 (7.3)
Decubitus Ulcer	137 (0.76)	72 (0.44)	24 (3.0)	41 (5.0)
***Selected Antibiotics, Previous 6 months***				
Beta-lactams	2,498 (13.9)	2,144 (13.1)	135 (17.0)	219 (26.7)
Fluoroquinolone	1,899 (10.5)	1,623 (9.9)	119 (15.0)	157 (19.2)
Topical (mupirocin)	135 (0.75)	95 (0.58)	12 (1.5)	28 (3.4)
MRSA Active Antibiotic	1,177 (6.5)	982 (6.0)	60 (7.6)	135 (16.5)
Any Antibiotic	5,311 (29.4)	4,678 (28.5)	260 (32.8)	373 (45.5)

### Carriage Patterns at 30 Days

The proportions of patients categorized as never, intermittently, or always colonized at the 30-day baseline were 91.1%, 4.4%, and 4.6%, respectively (Table 1). Patients in the never colonized group were less likely to be over 70 years of age (39%) than intermittently (47%) or always (54%) colonized patients (p <.001, Chi square for trend). A history of health care exposure in the past year, including acute or long-term care admissions, was more frequent in always colonized versus never colonized patients. A culture was positive for MRSA in the previous year in less than 1% of the total cohort and was present in 76 (9.3%) and 28 (3.5%) of patients in the always and intermittent carriage pattern groups, respectively. Co-morbidities including diabetes, liver disease, eczema, and decubitus ulcer were each significantly increased in always compared to intermittently and never colonized patients. A higher proportion of patients in the always colonized group had received an antibiotic in the previous 6 months, including MRSA active and non-active agents. Fluoroquinolones were used twice as frequently in always colonized compared with never colonized patients (Table 1).

In multivariate models, independent variables associated with MRSA nasal carriage (intermittent or always) compared to non-carriage at the 30-day baseline included advancing age, acute or long-term care admission in the past year, diabetes mellitus, renal disease (independent of dialysis), decubitus ulcer, and any antibiotic use in the past 6 months (Table 2). The presence of a decubitus ulcer was the strongest predictor of having a colonized carriage pattern (OR 5.33, 95% CI 3.71;7.66).

**Table 2:** Multivariable model of risk factors for patients with an always or intermittently colonized compared to a never colonized nasal MRSA carriage pattern at 30 days.

*Variable*	Odds Ratio	95% CI	P Value
**Age 70 or less**	0.61	(0.54; 0.68)	<.001
**Acute care/past year**	1.77	(1.56; 2.01)	<.001
**Long term care/past year**	2.82	(2.28;3.49)	<.001
**Diabetes**	1.22	(1.09;1.37)	<.001
**Renal Disease**	1.47	(1.22;1.77)	<.001
**Decubitus ulcer**	5.33	(3.71;7.66)	<.001
**Any antibiotic use/past 6 months**	1.35	(1.21;1.51)	<.001

### Performance of the 30-day PCR Carriage Pattern at 1 Year

There were 4,972 patients with a carriage pattern designated at 30 days who had 5 or more nasal screens for MRSA performed during the one year period from the index admission. Among the 4,415 patients with an initial non-colonized carriage pattern, 3,857 (87.4%) remained negative for nasal MRSA, 553 (12.5%) were intermittently MRSA nasal positive, and 5 (0.1%) developed persistent MRSA colonization. Thus, the 30-day non-colonized carriage pattern had a specificity of 50% (558/1115) compared to a 1-year pattern, but because of a high prevalence of the non-colonized state, the positive predictive value was 87% (3857/4415) for being non-colonized at one year. Movement from an intermittent carriage pattern to an always pattern occurred in 39/304 (12.8%) patients and from always (more than 80% of screens positive) to intermittent (less than 80% screens positive) in 80/253 (31.6%) of patients.

### Conversion from a Non-colonized Carriage Pattern

Among 4597 patients who were non-colonized at 30 days and had at least one re-screening performed in the following 6 months, 509 (11%) had a positive nasal PCR for MRSA. One to 25 screening tests were performed per person during the 6 months, with no difference in the mean number of tests between carriage patterns. An additional 33 (0.8%) patients had a conversion based on a positive culture for MRSA during the same time period. During the subsequent 6 months, an additional 8.5% of 2,496 patients with an initial non-colonized carriage pattern who were not identified as converting in the first 6 months became positive for MRSA. After adjusting for the number of swabs performed, number of long term care admissions, days of inpatient care, and co-morbidities, the predictors of conversion from never to a colonized pattern included age greater than 70 years, long term care admission in the previous year, and antibiotic use in the follow-up period (MRSA-active as well as other) (table 3). There was a trend for conversion in patients with diabetes (table 3). The presence of age greater than 70, diabetes, long term care admission in the year prior, or antibiotic use in the time from initial carriage pattern designation identified more than 95% of patients who were rescreened and converted over 6 months.

**Table 3:** Multivariable model of risk factors for conversion from a non-colonized nasal MRSA carriage pattern to colonized over the next 6 months.

*Variable*	Odds Ratio	95% CI	P value
**Age 70 or less**	0.66	(0.54;0.80)	<.001
**Number of swabs in follow-up period**	1.10	(1.06;1.14)	<.001
**Long term care/past year**	2.64	(1.73;4.04)	<.001
**Diabetes**	1.20	(0.98;1.46)	.08
**MRSA active antibiotic in follow-up period**	1.35	(1.11;1.65)	.003
**Other antibiotic in follow-up period**	1.57	(1.19;2.07)	.002

### Algorithm for Reducing Screening

**Figure 2:**
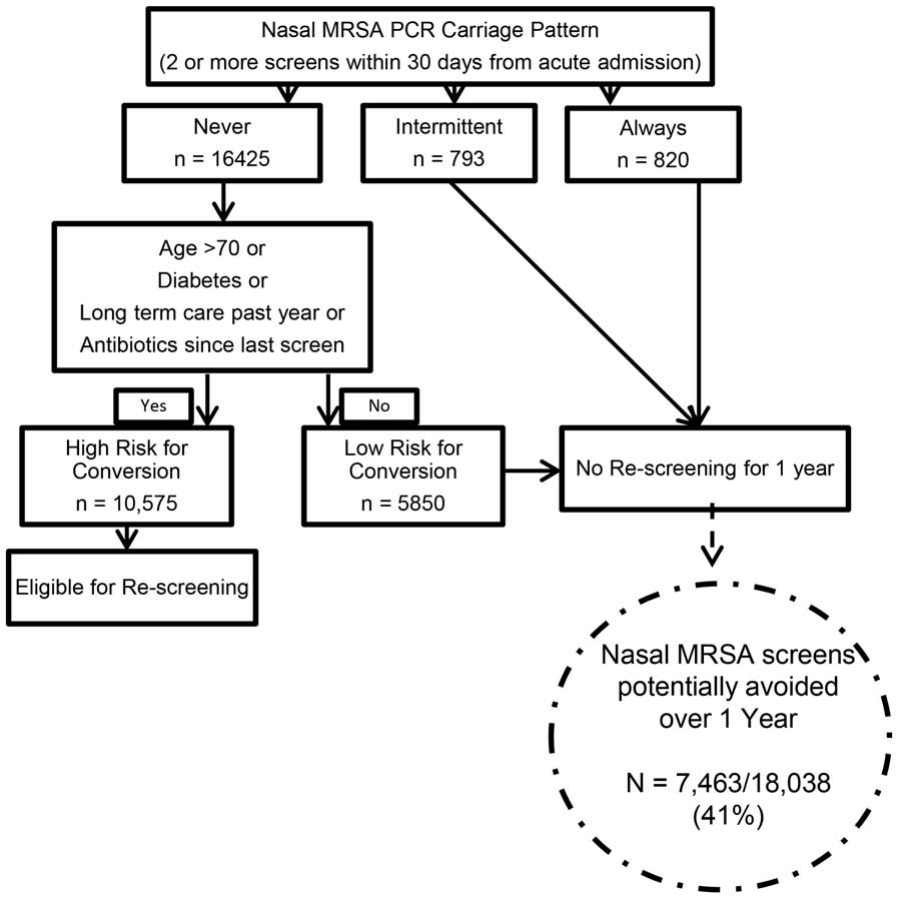
Proposed Algorithm for Reducing Screening. Number of nasal MRSA screens avoided by application of the initial carriage pattern and clinical criteria identifying patients at low risk for conversion.

Applying the 4 clinical criteria to the 16,425 patients who were not colonized at baseline identified 5,850 patients who comprised a low risk for conversion group (figure 2). Among this low risk group, 25 of 391 (6.4%) re-screened patients converted in the first 6 months and 36 of 698 (5.2%) converted in the second 6 months, for a total of 61 patients (5.7% of 1,089 re-screened patients and 1% of the whole low risk group) converting to MRSA positive over a one year period. Among patients with an initial non-colonized carriage pattern who were not in the low risk group and were re-screened, the rate of conversion was 11.5% in the first 6 months and 9.7% in the second 6 months, for an annual conversion of 10.9% among 6,236 re-screened patients or 6.4% among the entire “high risk” group. The number of screens that could be avoided if the 5,850 low risk and 1,613 already colonized (793 intermittent + 820 always) groups were not re-screened is 7,463 (41% of the original cohort; figure 2).

### Outcomes of Death and Infection

**Figure 3:**
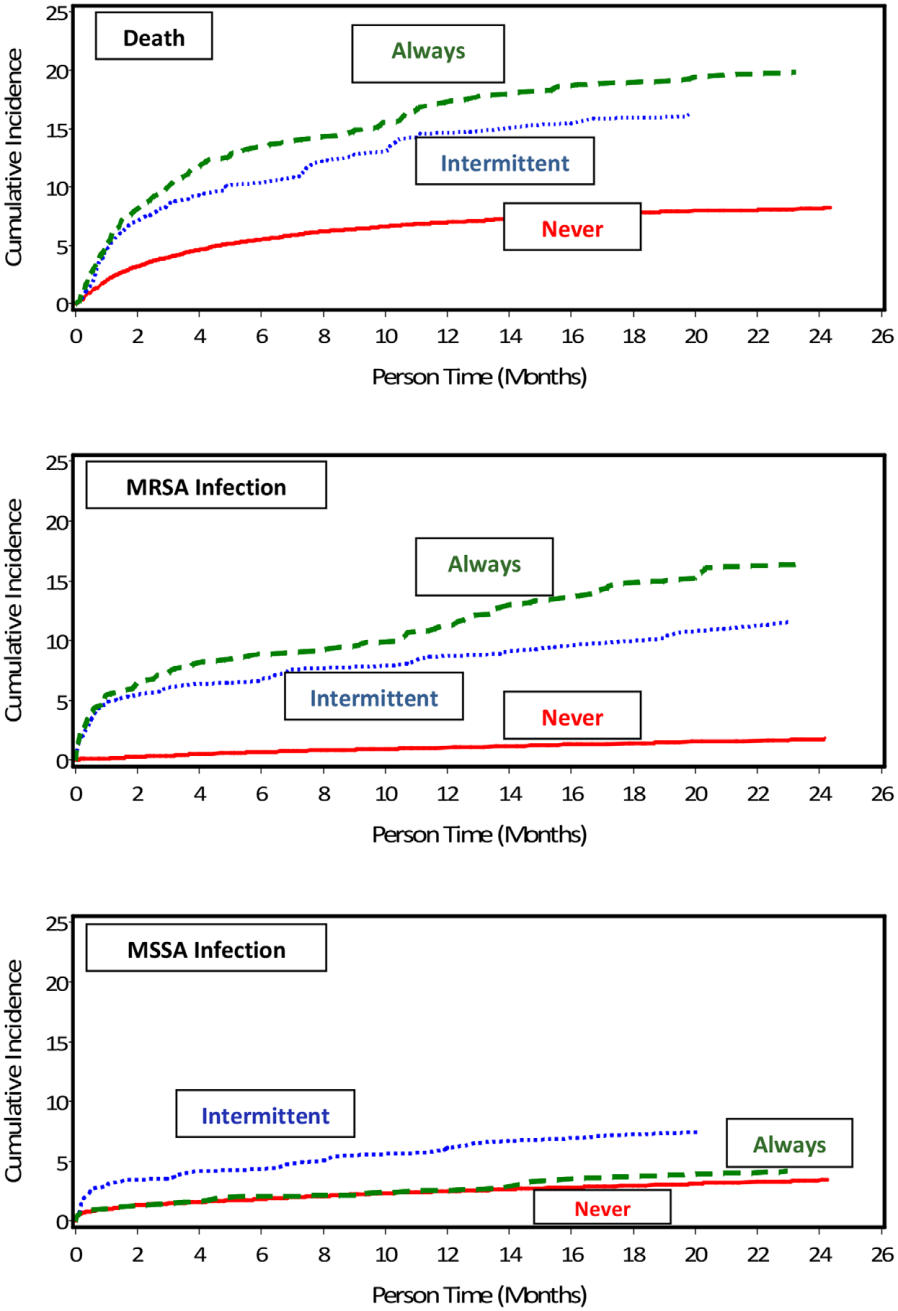
Outcomes by Carriage Pattern. The time to (a) death, (b) MRSA infection, and (c) MSSA infection in patients who had a non-colonized, intermittently colonized, or always colonized nasal carriage pattern at 30 days. The risk of death and of MRSA infection, adjusted for age, number of screening tests in the follow-up period, number of acute care and long term care admissions, total acute care and long term care hospital days, diabetes, renal disease, HIV infection, decubitus ulcer, eczema, and antibiotic exposure, was significantly higher among patients with a colonized compared to non-colonized nasal carriage pattern.

The outcomes of death and infection (MRSA and MSSA) were evaluated based on the 30 day carriage pattern as well as the dynamic carriage pattern over the study period. Death occurred in 1500 patients (8.4% of the study cohort), with an increasing rate with higher carriage burden at the 30-day baseline; 7.6% of never colonized, 15.0% of intermittently colonized, and 18.6% of always colonized patients died during follow-up. The mean follow-up time was 17.5, 16.5, and 17.2 months and the mean time to death from initial screening was 4.7, 4.5, and 4.9 months, respectively, in the never, intermittent and always colonized groups. Compared to non-colonized patients, those with intermittent or always colonized carriage patterns had a greater than 2-fold increased risk of death (HR 2.09; 95% CI 1.73;2.53 and HR 2.58; 95% CI 2.18;3.05, respectively) (figure 3a). After accounting for age, number of screening tests in the follow-up period, number of acute care and long term care admissions, total acute care and long term care hospital days, diabetes, renal disease, HIV infection, decubitus ulcer, eczema, and antibiotic exposure, the risk of death remained significantly elevated among always (HR 1.91; 95% CI 1.60;2.28) and intermittently colonized (HR 1.75; 95% CI 1.45;2.12) patients. When accounting for changes in carriage pattern over the study period (the dynamic carriage pattern), the adjusted risk of death was even higher among patients always colonized as compared to patients remaining non-colonized (HR 2.44; 95% CI 2.04;2.91).

There were 400 patients with an MRSA infection over the study follow-up period, with 221 (1.36%), 73 (9.48%), and 106 (13.43%) occurring in the non, intermittent, and always colonized 30-day carriage pattern groups. The 400 MRSA infections in unique patients occurred in blood (14.8%), bone (2.8%), abscess/fluid (26.5%), respiratory (38%), skin and soft tissue (16.5%), and other sites (1.3%). The mean follow-up time was 17.5, 15.2, and 15.6 months and the mean time to infection was 8.4, 5.0, and 6.1 months respectively among never, intermittently, and always colonized groups. The risk of infection was more than 10 fold greater among always colonized carriage pattern patients compared to the non-colonized carriage pattern (HR 10.89; 95% CI 8.6; 13.72) (figure 3b). After controlling for health care exposures and co-morbidities (same model as outlined above for death), the risk of MRSA infection remained elevated among always (HR 6.88; 95% CI 5.38;8.80) and intermittently (HR 5.46; 95% CI 4.17;7.14) colonized patients. Accounting for the dynamic carriage pattern (colonization occurring during follow-up), the adjusted risk of MRSA infection in the colonized group was 17 fold higher compared to those that remained in the non-colonized carriage group (HR 17.02; 95% CI 12.91;22.44).

MSSA infections occurred in 536 patients and were most common in the intermittent MRSA carriage pattern group (6.3%) compared to the always (3.2%) and non-colonized (2.9%) groups. The risk of infection with MSSA was significantly higher in intermittently colonized as compared to non-colonized patients (HR 2.36; 95% CI 1.76;3.16) but not in patients who were persistently colonized with MRSA (figure 3c). In multivariable analysis, the adjusted risk of MSSA infection remained significantly elevated in patients who were intermittently colonized with MRSA (HR 1.84; 95% CI 1.37;2.48). The adjusted risk of MSSA infection was not significantly associated with patterns of nasal MRSA carriage when accounting for the changes in carriage over the study period.

## Discussion

The VA MRSA Prevention Initiative, a bundle which includes both horizontal and vertical measures, has been temporally associated with significant reductions in hospital-associated MRSA infections [9]. Universal active surveillance is a costly and resource-intensive component of the program [11]. Some studies have tried to identify a sub-group of patients who are at high risk of being colonized to help develop targeted screening programs. However, most studies have found that a risk-based approach has low sensitivity, missing at least 25–30% of patients who are later found to be colonized [12]. In the absence of robust algorithms to support high-risk screening, universal surveillance has continued.

We employed a novel approach by identifying initial patterns of nasal MRSA carriage and then developing an algorithm to reduce screening based on eliminating further screens on patients already colonized as well as those at low risk for conversion. Given that the average prevalence of MRSA colonization on hospital admission to an acute care VA hospital is 13%, the non-colonized group represents the vast majority of hospitalized veterans [9]. Our study demonstrates that only 11.8% of patients who have two or more consecutive nasal PCR swabs that are negative for MRSA in a 30-day period have a subsequent positive culture or swab result in the following 6 months, despite re-admission to the hospital. The rate of conversion over the next 6-month period is even lower, with an additional 8.5% of screened patients becoming positive for MRSA. Importantly, the conversion rates among the low risk sub-group were only 5–6% over the same 6 month periods. Thus the vast majority of patients at risk for becoming colonized with MRSA can be identified based on their initial nasal carriage pattern and four clinical criteria.

Patients who have a positive carriage pattern on the initial hospitalization continue to carry MRSA either always or intermittently over long periods of time [13]. The benefit of repeated MRSA screening in these patients is unclear, since most will not have the consistently negative result over 3 consecutive tests in the absence of antibiotic exposure that would be required to move the patient out of the “MRSA-colonized” category. Annual repeat assessments may be reasonable for assessing changes in carriage pattern, particularly if MRSA colonization risk factors such as antibiotic use are absent.

Future screening and infection prevention efforts should focus on the group of patients with an initial negative MRSA carriage pattern who do not meet criteria for being at low-risk for MRSA conversion. Based on our results, patients who are in long term care, greater than 70 years of age, have diabetes or antibiotic use are at highest risk of conversion to a MRSA colonized pattern over a 6 month period. These criteria identified 95% of patients who converted from non-colonized to colonized in our cohort and could be applied at the time of admission to help direct screening. Although antibiotic use may be more reliably captured in health care systems with robust and integrated electronic health records, a four point algorithm is less complex than what other studies have proposed and could be applied at the bedside [14]. If additional surveillance tests were avoided for a year on patients who at 30 days were found to be always or intermittently colonized and on patients who were not colonized and at low risk for conversion, approximately 7,500 surveillance tests could be spared at the VISN level. At an estimated cost of $30–$50 per test, this could result in significant annual cost savings within our VISN ($300–400 K) and nationally (millions) without adversely affecting the main goal of active surveillance, detection of colonized patients not known to have MRSA [11], [15]. Other facilities considering active MRSA nasal screening on admission due to financial pressure to reduce in-hospital acquisition could potentially reduce their screening burden by using this clinical tool. However, future studies will be needed to evaluate generalizability to non-VA settings and the feasibility and efficacy of implementing a testing reduction tool.

Our study demonstrates that establishing a carriage pattern of MRSA colonization is useful not only for developing targeted screening programs, but also for predicting the risk of death and MRSA infection. Previous studies have found that MRSA carriage is a risk factor for subsequent infection, both in the immediate period after acquisition of MRSA as well as among patients who are carriers for over a year [4], [8]. Most of these studies identified patients based on cultures that were obtained for clinical purposes rather than via active nasal screening. In one study of 591 patients newly detected to have MRSA (94% had an infection or clinical culture as the index event) on admission to a tertiary care center, 33% developed an MRSA infection in the following year. Thus, clearly, among patients with a new MRSA infection or positive clinical culture, subsequent infection and mortality rates are high. Our data add to this concept by defining an infection and death risk among patients with asymptomatic nasal colonization, both of which occurred in a linearly increasing frequency with an increasing burden of colonization (non-colonized, intermittent, and always colonized patterns). The risk of infection in particular markedly increased as the carriage pattern changed over the follow-up period, with a more than double increased risk ratio in patients who became colonized during follow-up. This association warrants further evaluation for potentially modifiable pathways. Whether strategies other than simple detection and isolation would be useful in reducing infection risk in these patients remains an area of active investigation.

The main limitation of our study is the potential misclassification of patients who had fewer screening tests (less chance of converting carriage pattern). To minimize bias in our findings, our models are adjusted for the number of screening tests performed, and we also did not perform a time to event analysis of conversion since it greatly depended on repeat testing rather than a clinically apparent event such as death or infection. The findings are derived from a demographic representative of the Veteran population and thus may not translate fully to other populations. The performance of the reduced screening strategy based on carriage pattern and risk for conversion merits continued evaluation over time and in additional populations. We used an electronic definition of infection that may have misclassified some positive cultures in either direction. The infection rates generated using this electronic definition, however, are similar to those reported by another study that used a chart review-based infection definition [4]. Nasal only screening could have missed some people who were carriers of MRSA, but the rate of exclusive extra-nasal colonization is low in this population [16], [17]. We also did not account for MSSA colonization or differences in virulence among nasal MRSA isolates that could have impacted infection risk. Our recent molecular evaluation of nasal MRSA and MSSA among newly colonized veterans identified a low prevalence of USA300 and Panton-Valentine Leukocidin (PVL) genes and no association between USA300 MRSA carriage and subsequent infection risk [13].

The greatest strength of our study compared to previous studies of infection risk in colonized patients is the ability to capture data from all inpatient and outpatient encounters at multiple institutions over a broad geographic region over a 3 year period and define patterns of nasal carriage rather than rely on a single assessment. The VA is the largest integrated health care system in the country with a robust electronic health record system, enhancing the detection of events occurring over the continuum of care, including all VA acute care hospitals in the region, long-term care facilities, and the outpatient setting. Our data also have the advantage of representing the real-practice setting, without experimental interventions that may or may not be feasible within current health care delivery systems. Also, we were not reliant on ICD-9 coding for MRSA infections, which has been shown to be a poor measure in VA-based studies [18].

### Conclusions

The MRSA nasal carriage pattern based on at least 2 nasal PCR screens within 30 days in acutely hospitalized patients is a strong predictor of ongoing carriage (and non-carriage), MRSA infection, and all-cause mortality. Our findings have significant implications for future policy considerations regarding the highly successful VA MRSA Prevention Initiative, providing evidence that one of the most costly components, universal active surveillance, could be targeted towards the group of patients most likely to convert from negative to positive over a one year period. The effectiveness and optimal implementation of this strategy needs to be studied along with continued assessment of additional activities aimed at reducing subsequent infections.
